# Assessing artificial intelligence in breast screening with stratified results on 306 839 mammograms across geographic regions, age, breast density and ethnicity: A Retrospective Investigation Evaluating Screening (ARIES) study

**DOI:** 10.1136/bmjhci-2024-101318

**Published:** 2025-05-14

**Authors:** Cary J G Oberije, Rachel Currie, Alice Leaver, Alan Redman, William Teh, Nisha Sharma, Georgia Fox, Ben Glocker, Galvin Khara, Jonathan Nash, Annie Y Ng, Peter D Kecskemethy

**Affiliations:** 1 Kheiron Medical Technologies Limited, London, Greater London, UK; 2 Breast Screening Programme, Royal Devon and Exeter NHS Foundation Trust, Exeter, Devon, UK; 3 Breast Screening Unit, Gateshead Health NHS Foundation Trust, Gateshead, Gateshead, UK; 4 Breast Screening Programme, Royal Free London NHS Foundation Trust, London, UK; 5 Breast Screening Programme, Leeds Teaching Hospitals NHS Trust, Leeds, UK; 6 Department of Computing, Imperial College London, London, UK; 7 DeepHealth, Somerville, MA, USA

**Keywords:** Artificial intelligence, Health Equity, Primary Health Care

## Abstract

**Objectives:**

Evaluate an Artificial Intelligence (AI) system in breast screening through stratified results across age, breast density, ethnicity and screening centres, from different UK regions.

**Methods:**

A large-scale retrospective study evaluating two variations of using AI as an independent second reader in double reading was executed. Stratifications were conducted for clinical and operational metrics. Data from 306 839 mammography cases screened between 2017 and 2021 were used and included three different UK regions.

The impact on safety and effectiveness was assessed using clinical metrics: cancer detection rate and positive predictive value, stratified according to age, breast density and ethnicity. Operational impact was assessed through reading workload and recall rate, measured overall and per centre.

Non-inferiority was tested for AI workflows compared with human double reading, and when passed, superiority was tested. AI interval cancer (IC) flag rate was assessed to estimate additional cancer detection opportunity with AI that cannot be assessed retrospectively.

**Results:**

The AI workflows passed non-inferiority or superiority tests for every metric across all subgroups, with workload savings between 38.3% and 43.7%. The AI standalone flagged 41.2% of ICs overall, ranging between 33.3% and 46.8% across subgroups, with the highest detection rate for dense breasts.

**Discussion:**

Human double reading and AI workflows showed the same performance disparities across subgroups. The AI integrations maintained or improved performance at all metrics for all subgroups while achieving significant workload reduction. Moreover, complementing these integrations with AI as an additional reader can improve cancer detection.

**Conclusion:**

The granularity of assessment showed that screening with the AI-system integrations was as safe as standard double reading across heterogeneous populations.

**WHAT IS ALREADY KNOWN ON THIS TOPIC**

- Retrospective and prospective studies have shown that integrating Artificial Intelligence (AI) in double reading workflows for breast screening can maintain or improve performance at the population level. However, information on subgroup performance is largely lacking.

**WHAT THIS STUDY ADDS**

- Patient characteristics are linked to biomedical features that influence cancer prevalence as well as detection accuracy. Human double reading thus shows differences in outcome metrics for different patient subgroups. This study shows that workflows where AI has been integrated into double reading do not exacerbate such performance disparities.

**HOW THIS STUDY MIGHT AFFECT RESEARCH, PRACTICE OR POLICY**

- This study shows that integrating AI can maintain or improve clinical outcomes for participants of different age, breast density and ethnicity across different regions while providing significant workload savings for sites. In addition, the AI detected a large proportion of interval cancers, offering a possibility to increase cancer detection rates.

## Introduction

 Many retrospective studies have demonstrated the potential of Artificial Intelligence (AI) to support the effectiveness and sustainability of breast screening by decreasing radiologists’ workload while maintaining or improving screening performance.^1 2^ This has led to calls for prospective, real-world evaluations of AI, with highly encouraging early results.^3 4^ However, current evidence is still lacking in adequate assessments to ensure safety and effectiveness across relevant patient subgroups.

While having clinical evidence on whole populations is important to determine overall effectiveness, detailed stratified analyses across relevant subgroups are required to ensure no undue harm is caused to a particular subgroup, which might be masked by an overall improved outcome.^5 6^ Biases present in the data used to develop AI may be reflected in the resulting model and might systematically reduce performance on some subgroups in live use.^7^

Prospective evaluations can explore the impact of AI in clinical practice and are needed to confirm and accurately quantify the benefits of AI deployments. Recent prospective evaluations have demonstrated a significant cancer detection increase possible, ranging from 0.7 to 1.6 per thousand.^3 4^ However, prospective studies are usually too limited in sample size for adequate subgroup analyses. Large-scale retrospective studies, on the other hand, enable a granular evaluation across patient subgroups, especially in breast screening where cancer prevalence is low, due to their large size—while their ability to explore improvements in cancer detection is limited.^8^ Consequently, both are required, providing different aspects of assessment, on the journey of validating, deploying and adopting effective AI safely.

The interpretation of subgroup results is not straightforward. Some patient characteristics are linked to biomedical features, resulting in differences in disease prevalence or complicating disease detection, such as breast density.^9^,^11^ Consequently, human reading without AI inherently exhibits differences in screening performance across subpopulations.^12 13^ Human and AI workflows’ performance should not be expected to deliver equal care outcomes across different participant groups, as that may fundamentally not be possible. One of the criteria to support safety and effectiveness for AI is that AI deployments should not cause systematically lowered performance for subgroups compared with standard of care.

In A Retrospective Investigation Evaluating Screening (ARIES) study – a large-scale, multicentre retrospective study – two independent reader AI workflow variants are modelled and assessed for clinical and operational impact.^2 14^ These independent reader AI-workflows are primarily intended to provide workload savings and are expected to complement other AI workflows that support increased cancer detection, such as AI as an additional reader.^4^ Relevant screening performance metrics are evaluated across multiple centres from different UK regions, participant age, breast density and ethnicity, demonstrating the strength and critical role of large-scale retrospective studies in assessing the safety and effectiveness of the use of cancer detection AI.

## Methods

### Study population and data

The study included data from three centres, specifically selected from UK regions with different dominant genetic backgrounds,^15^ including the Gateshead Breast Screening Programme (Gateshead), InHealth’s North & East Devon Breast Screening Programme (NED) and Royal Free London NHS Foundation Trust’s North London Breast Screening Programme (RFL). Data including participant age, ethnicity and cancer outcomes were de-identified and collected from the National Breast Screening System (NBSS). Ethnicity information was used from only RFL where ethnicity is collected as part of their standard procedure. Breast density Breast Imaging-Reporting and Data System (BI-RADS) classification was automatically generated using an AI breast density estimation tool (DeepHealth).^16^

The study included screening cases from 2017 to 2021 that were compatible with the evaluated AI system and had exactly four-view full-field digital mammograms (FFDMs), with all human reader recall/no recall opinions available.

### Patient and public involvement

The development and final design of the study protocol was reviewed with Kheiron’s Patient and Public Involvement Advisory Board. As this was a retrospective study, no patients were involved in the recruitment and conduct of the study. Results will be available and disseminated to study patients through scientific academic publications and the study trial registration.

### Standard double reading

In standard double reading (DR), each case is initially evaluated by two human readers who each decide whether or not to recall a woman for further assessment. If the readers disagree, an arbitration of the case will take place to reach a final decision. If the readers agree no recall, no further action is required. If the readers agree to recall for further assessment, depending on the site’s procedure, the woman is either directly recalled for further assessment or a final decision will be made after an arbitration procedure.

### Artificial Intelligence (AI) system

The commercially available AI system (DeepHealth) is a combination (ensemble) of multiple models including a diverse set of different deep convolutional neural networks. The AI system was developed with patient safety and fairness in mind, using a heterogeneous, large-scale collection of data from real-world screening programmes across different countries, a multitude of centres and with equipment from different mammography hardware vendors over a period of more than 10 years.^2^

The system works with standard Digital Imaging and Communications in Medicine images as inputs. It analyses four images with two standard FFDM views (craniocaudal and mediolateral oblique) per breast. The primary output of the AI system is a case-wise binary recommendation of recall (for further assessment due to suspected malignancy) or no recall (until the next screening interval). No data from the study centres were used in the development or training of the AI system’s machine learning model. The AI system’s software version and operating points (OP) used in the ARIES study were fixed prior to its analysis of any study data. See the online supplemental methods for further detail.

### Artificial Intelligence (AI) workflows

The AI system can be used in different and multiple roles in breast screening. Two variants of DR workflows with AI as an independent reader, primarily intended to provide operational benefits, were evaluated and compared against standard human DR (online supplemental figure 1). In one variant, called ‘supporting independent reader (sIR)’, the AI serves as the second reader in cases where it agrees with the first human reader (R1) to recall or not recall a case.^2 14^ In a second variant, ‘double reader triage (DRT)’, the AI serves as the second reader in cases where it agrees with R1 to not recall a case.^2 14^ In both versions, when there is disagreement between R1 and AI, the case is deferred to a second human reader, defaulting to standard DR. These workflows were assessed at multiple pre-set OP-configurations, but only a single configuration is presented per AI-workflow that is intended to provide a balance between clinical and operational benefits.

### Outcome metrics

The metrics assessed to inform clinical safety and effectiveness on screening participants across relevant participant subgroups—age, breast density and ethnicity—were cancer detection rate (CDR) to characterise early cancer detection and positive predictive value (PPV) to ensure false-positive recalls are minimised, as they could lead to participant anxiety^17^ and reduced subsequent screening uptake.^18^ Metrics that inform operational efficiency and sustainability for screening services were assessed per centre, including human reading workload and recall rate (RR) as a measure for downstream impact of further assessments. Sensitivity (SEN) and specificity (SPEC) were also assessed as common metrics used to evaluate diagnostic tools. Arbitration rate (AR) was assessed as it is a component of total workload. Cancers included biopsy or histopathology confirmed screen-detected and interval cancers (IC). Metric calculations are described in the online supplemental file 1.

The portion of IC cases flagged as suspicious by the AI system was calculated as a proxy measure for additional cancer detection possible using the AI as an additional reader (XR) to flag cases not recalled by DR for additional review. While it is not possible to assess the additional CDR detection of the XR workflow in a retrospective setting, the IC flag rate of the AI system can be used to estimate what could be achieved in a prospective setting. A comprehensive explanation of the method is in the online supplemental file 1.

### Statistical analysis

Patient characteristics were reported and differences between centres were tested using analysis of variance (ANOVA) or χ2 tests, depending on the data type. An overall test was performed with an alpha of 0.05. Only if this test was statistically significant, subsequent tests were carried out, comparing all combinations of the three sites, using a Bonferroni correction for the p-value.

All outcome metrics were calculated at predetermined AI OP. Calculations are reported with two-sided 95% CIs, based on Wilson Scores. The overall outcome metrics were calculated as the equally weighted mean of the three centres, to ensure that each centre’s influence on the overall statistic was equal. Non-inferiority tests were performed to compare the AI workflows to standard human DR using the bootstrap method. All tests were one-sided and used a relative non-inferiority margin of 5% and an alpha of 0.05. When non-inferiority passed, a superiority test was also performed with an alpha of 0.05. Further details can be found in the online supplemental file 1.

The percentage of AI-flagged IC cases was reported to indicate the opportunity for increased cancer detection. By using data from two centres, not included in the current study, where retrospective as well as prospective datasets were available, it was possible to estimate an expected CDR increase, based on the IC flag rate (online supplemental methods).

## Results

### Cohort characteristics

After applying the study eligibility criteria, the final study dataset consisted of 306 839 cases from 236 739 women. The Standards for Reporting of Diagnostic Accuracy Studies diagram is shown in online supplemental figure 2. Cohort characteristics are shown in table 1. χ2 tests for the categorical variables and the ANOVA test for age resulted in statistically significant differences. All subsequent tests to identify which centres differed from each other also showed significant differences.

**Table 1:** Cohort characteristics

		All ARIES centres	RFL	NED	Gateshead	
		306 839	189 257	65 839	51 743	
Variable	Value	N (%)	N (%)	N (%)	N (%)	P*
Age	Mean (SD)	59 (SD 7.1)	59.0 (SD 7.1)	60.7 (SD 7.2)	60.2 (SD 6.7)	<0.001
Age	≤49 year	13 403 (4.4%)	12 135 (6.4%)	798 (1.2%)	470 (0.9%)	<0.001
	50–59 year	149 623 (48.8%)	93 697 (49.5)	29 928 (45.5%)	25 998 (50.2%)	
	60–70 year	126 068 (41.1%)	73 032 (38.6)	29 997 (45.6%)	23 039 (44.5%)	
	≥71 year	17 745 (5.8%)	10 393 (5.5%)	5116 (7.8%)	2236 (4.3%)	
Breast density	A	38 905 (12.7%)	21 836 (11.5%)	8877 (13.5%)	8192 (15.8%)	<0.001
	B	139 407 (45.4%)	85 465 (45.2%)	30 119 (45.7%)	23 823 (46.0%)	
	C	114 869 (37.4%)	72 333 (38.2%)	24 336 (37.0%)	18 200 (35.2%)	
	D	13 306 (4.3%)	9410 (5.0%)	2505 (3.8%)	1391 (2.7%)	
	Missing	352 (0.1%)	213 (0.1%)	2 (0.003%)	137 (0.3%)	
Ethnicity	White	92 644 (30.2%)	92 644 (49.0%)	–	–	–
	Non-White†	65 060 (21.2%)	65 060 (34.4%)	–	–	–
	Asian	33 505 (10.9%)	33 505 (17.7%)	–	–	–
	Black	19 273 (6.3%)	19 273 (10.2%)	–	–	–
	Mixed/other	12 282 (4.0%)	12 282 (6.5%)	–	–	–
	Missing	149 135 (48.6%)	31 553 (16.7%)	–	–	–
Screening year	2017	66 341 (21.6%)	43 472 (23.0%)	14 132 (21.5%)	8737 (16.9%)	<0.001
	2018	69 012 (22.5%)	42 316 (22.4%)	14 435 (21.9%)	12 261 (23.7%)	
	2019	74 858 (24.4%)	47 711 (25.2%)	14 910 (22.6%)	12 237 (23.6%)	
	2020	31 806 (10.4%)	17 755 (9.4%)	9231 (14.0%)	4820 (9.3%)	
	2021	64 822 (21.1%)	38 003 (20.1%)	13 131 (19.9%)	13 688 (26.5%)	
Positives‡	Screen-detected cancer	2592 (0.8%)	1673 (0.9%)	577 (0.9%)	342 (0.7%)	<0.001
	Interval cancer	377 (0.1%)	158 (0.1%)	141 (0.2%)	78 (0.2%)	

* P values for post hoc χ2 and ANOVA tests, with Bonferroni corrected p values, comparing each site to the others, resulted in significant differences for all tests.

† Non-white is a combination of Black, Asian and Mixed/other ethnicity.

‡ The set of screen-detected and interval cancers are not mutually exclusive; two cases included in the screen-detected cancer set also had an interval cancer.

ANOVA, analysis of variance; ARIES, A Retrospective Investigation Evaluating Screening; Gateshead, Gateshead Breast Screening Programme; NED, InHealth’s North & East Devon Breast Screening Programme; RFL, Royal Free London NHS Foundation Trust’s North London Breast Screening Programme.

The majority of eligible cases were from RFL (61.7%; 1 89 257 cases), where ethnicity information was available for analysis. 65 839 cases (21.5%) and 51 743 cases (16.9%) were eligible from NED and Gateshead, respectively. The screening volumes at all centres were lower during the year of 2020 due to the COVID-19 pandemic.

A larger proportion of cases were screened for women less than 49 years of age at RFL and NED, which is in alignment with their participation in the AgeX trial. The higher percentage of women older than 71 years at NED was due to a higher self-referral rate.

For the overall study, most women were assessed as having breast density B (45.4%) or C (37.4%), while 12.7% were assigned category A and 4.3% category D.

### Results comparing Artificial Intelligence (AI) workflows with standard human double reading (DR)

For the overall study, non-inferiority tests comparing the AI workflows sIR and DRT to standard DR passed for CDR, while superiority tests passed for PPV and RR, next to substantial workload savings of 42.5% and 39.6% estimated for sIR and DRT, respectively (table 2).

**Table 2:** Overall results for the ARIES study on 306 839 cases

Assessment	Metric	Standard human double reading (DR)	Supporting independent reader (sIR)			Double reader triage (DR)		
		Value (95% CI)	Value (95% CI)	Absolute difference	Test passed	Value (95% CI)	Absolute difference	Test passed
Clinical (participant) impact	CDR (per 1000)	8.1 (7.8 to 8.5)	8.0 (7.7 to 8.3)	−0.12	Non-inferiority	8.0 (7.7 to 8.4)	−0.11	Non-inferiority
	PPV (%)	18.6 (18.0 to 19.2)	19.5 (18.8 to 20.1)	+0.85	Superiority	20.0 (19.3 to 20.7)	+1.4	Superiority
Operational (service) impact	RR (%)	4.4 (4.4 to 4.5)	4.1 (4.1 to 4.2)	−0.28	Superiority	4.1 (4.0 to 4.1)	−0.37	Superiority
	Workload (%)	100	57.5	−42.5	Not tested	60.4	−39.6	Not tested

ARIES, A Retrospective Investigation Evaluating Screening; CDR, cancer detection rate; PPV, positive predictive value; RR, recall rate.

Standard human DR demonstrated higher CDR for the older age group (10.2 vs 6.2 per 1000), high density breast group (8.9 vs 7.6 per 1000) and for White vs Non-White ethnicities at 9.9 per 1000 versus 8.0 per 1000. While PPV was higher for the older age group (26.2% vs 12.8%), it was lower for patients with high density breasts (17.6% vs 19.5%) and for Non-white versus White ethnicity (17.5% vs 19.6%). Both sIR and DRT closely followed the DR CDR and PPV patterns (table 3).

**Table 3:** Results for clinical metrics per subgroup: cancer detection rate and positive predictive value

Subgroup		Standard human double reading (DR)	Supporting independent reader (sIR)			Double reader triage (DRT)		
			Value (95% CI)	Absolute difference	Test passed*	Value (95% CI)	Absolute difference	Test passed*
Cancer detection rate (CDR) per 1000								
Centre	RFL	8.9 (8.5–9.3)	8.7 (8.3 to 9.1)	−0.19	Non-inferiority	8.7 (8.3 to 9.2)	−0.16	Non-inferiority
	NED	8.9 (8.2–9.6)	8.8 (8.1 to 9.6)	−0.08	Non-inferiority	8.8 (8.1 to 9.6)	−0.08	Non-inferiority
	Gateshead	6.6 (6.0–7.4)	6.5 (5.9 to 7.3)	−0.10	Non-inferiority	6.5 (5.9 to 7.3)	−0.10	Non-inferiority
Age	<60 years	6.2 (5.8–6.6)	6.1 (5.7 to 6.5)	−0.08	Non-inferiority	6.0 (5.7 to 6.4)	−0.11	Non-inferiority
	≥60 years	10.2 (9.7–10.7)	10.0 (9.5 to 10.6)	−0.16	Non-inferiority	10.1 (9.6 to 10.6)	−0.11	Non-inferiority
Breast density	A/B	7.6 (7.2–8.0)	7.5 (7.1 to 7.9)	−0.13	Non-inferiority	7.5 (7.1 to 7.9)	−0.09	Non-inferiority
	C/D	8.9 (8.4–9.4)	8.8 (8.3 to 9.3)	−0.10	Non-inferiority	8.7 (8.2 to 9.3)	−0.14	Non-inferiority
Ethnicity	White	9.9 (9.3–10.5)	9.6 (9.0 to 10.3)	−0.24	Non-inferiority	9.7 (9.1 to 10.3)	−0.21	Non-inferiority
	Non-White†	8.0 (7.4–8.7)	7.9 (7.3 to 8.6)	−0.12	Non-inferiority	7.9 (7.3 to 8.6)	−0.11	Non-inferiority

Positive predictive value (PPV) (%)								
Centre	RFL	17.7 (16.9–18.5)	19.1 (18.3 to 20.0)	+1.4	Superiority	19.1 (18.2 to 19.9)	+1.4	Superiority
	NED	17.9 (16.7–19.3)	19.1 (17.7 to 20.5)	+1.1	Superiority	19.7 (18.3 to 21.2)	+1.7	Superiority
	Gateshead	20.2 (18.3–22.2)	20.1 (18.3 to 22.1)	−0.05	Non-inferiority	21.1 (19.2 to 23.2)	+1.0	Superiority
Age	<60 years	12.8 (12.1–13.5)	13.4 (12.7 to 14.2)	+0.60	Superiority	13.7 (13.0 to 14.5)	+0.90	Superiority
	≥60 years	26.2 (25.1–27.4)	27.4 (26.2 to 28.6)	+1.2	Superiority	28.1 (26.9 to 29.3)	+1.9	Superiority
Breast density	A/B	19.5 (18.6–20.4)	20.5 (19.6 to 21.5)	+1.0	Superiority	21.0 (20.0 to 22.0)	+1.5	Superiority
	C/D	17.6 (16.7–18.5)	18.2 (17.3 to 19.2)	+0.65	Superiority	18.8 (17.9 to 19.8)	+1.2	Superiority
Ethnicity	White	19.6 (18.5–20.7)	21.1 (19.9 to 22.4)	+1.6	Superiority	21.1 (19.9 to 22.3)	+1.5	Superiority
	Non-White†	17.5 (16.2–18.9)	19.0 (17.6 to 20.5)	+1.5	Superiority	18.9 (17.4 to 20.4)	+1.4	Superiority

* One-sided tests are based on ratios, an alpha of 0.05 and a non-inferiority margin of 5%.

† Non-White is a combination of Black, Asian and mixed/other ethnicity.

Gateshead, Gateshead Breast Screening Programme; NED, InHealth’s North & East Devon Breast Screening Programme; RFL, Royal Free London NHS Foundation Trust’s North London Breast Screening Programme.

Clinical impact comparison of sIR and DRT to standard DR demonstrated superior PPV across all centres and the age, breast density and ethnicity subgroups, with point estimates ranging from an absolute 0.60% to 1.9% increase, apart from the test conducted for the Gateshead screening centre for sIR (table 3). The non-inferiority test on CDR passed for the AI workflows compared with DR, ranging from an absolute reduction of −0.24 to −0.08 per 1000 across all centres and subgroups (table 3). Among the absolute differences calculated between sIR and DRT to DR per participant subgroup, the maximum difference between each binary subgroup is 0.12 per 1000 for CDR and 1.0% for PPV.

The operational impact results for sIR and DRT compared with DR demonstrated an absolute reduction in RR ranging from −0.48% to −0.04%, passing non-inferiority and superiority tests overall for the study and each centre (table 4). For sIR, the estimated total workload savings were measured as 43.7%, 42.9% and 41.0% for Gateshead, NED and RFL, respectively. For DRT, the workload savings were 41.0%, 39.6% and 38.3% for Gateshead, NED and RFL, respectively (table 4).

**Table 4:** Results for operational metrics overall and per centre: recall rate and workload

Subgroup	Standard human double reading (DR)	Supporting independent reader (sIR)			Double reader triage (DRT)		
	Value (95% CI)	Value (95% CI)	Absolute difference	Test passed*	Value (95% CI)	Absolute difference	Test passed*
Recall rate (RR) (%)							
Overall	4.4 (4.4 to 4.5)	4.1 (4.1 to 4.2)	−0.28	Superiority	4.1 (4.0 to 4.1)	−0.37	Superiority
RFL	5.0 (4.9 to 5.1)	4.5 (4.5 to 4.6)	−0.48	Superiority	4.6 (4.5 to 4.7)	−0.44	Superiority
NED	5.0 (4.8 to 5.1)	4.6 (4.5 to 4.8)	−0.34	Superiority	4.5 (4.3 to 4.6)	−0.48	Superiority
Gateshead	3.3 (3.1 to 3.4)	3.2 (3.1 to 3.4)	−0.04	Non-inferiority	3.1 (2.9 to 3.2)	−0.20	Superiority

Workload compared with DR (%)							
Overall	100	57.5	−42.5	Not tested	60.4	−39.6	Not tested
RFL	100	59.0	−41.0	Not tested	61.7	−38.3	Not tested
NED	100	57.1	−42.9	Not tested	60.4	−39.6	Not tested
Gateshead	100	56.3	−43.7	Not tested	59.0	−41.0	Not tested

* One-sided tests are based on ratios, an alpha of 0.05 and a non-inferiority margin of 5%.

DR, standard human double reading; Gateshead, Gateshead Breast Screening Programme; NED, InHealth’s North & East Devon Breast Screening Programme; RFL, Royal Free London NHS Foundation Trust’s North London Breast Screening Programme.

As expected, sIR provided higher workload savings than DRT, while DRT resulted in a higher reduction in RR and higher increase in PPV compared with sIR, with its focus on reducing recalls. See online supplemental file 1 for the detailed results for AR, SEN and SPEC.

The AI system in a standalone capacity flagged 41.2% of the screening cases of ICs as suspicious. The portion of ICs flagged ranged between 33.3% and 46.8% across the centres and age, breast density and ethnicity subgroups (table 5). The IC rate was highest in the density C/D subgroup, for which the AI system’s IC flag rate was also the highest (46.8%) (table 5).

**Table 5:** Interval cancer (IC) case flag rate

Subgroup	Portion IC cases flagged (%)*	IC rate in group (per 1000)	Total cases in group	Number of ICs in group	Number of IC cases flagged
Overall					
All centres	41.2 (36.3, 46.2)†	1.2	306 839	379	152

Per centre					
Gateshead	46.8 (36.2, 57.7)	1.5	51 743	79	37
NED	39.4 (31.8, 47.7)	2.2	65 839	142	56
RFL	37.3 (30.2, 45.1)	0.8	189 257	158	59

Per age					
<60 year	38.8 (32.8, 45.6)	1.3	163 026	218	80
≥60 year	45.1 (37.8, 53.1)	1.1	143 813	161	72

Per breast density					
A/B	33.3 (25.3, 41.8)	0.7	178 312	121	40
C/D	45.2 (39.4, 51.4)	2.0	128 175	258	112

Per ethnicity					
White	37.6 (28.5, 47.8)	1.0	92 644	93	35
Non-White‡	37.0 (24.5, 51.4)	0.7	65 060	46	17

* 95% CIs are in parentheses.

† Metric was calculated as the equally weighted mean of the three centres, to ensure that each centre’s influence on the overall statistic was equal. See statistical analysis section of methods.

‡ Non-White is a combination of Black, Asian and mixed/other ethnicity.

Gateshead, Gateshead Breast Screening Programme; NED, InHealth’s North & East Devon Breast Screening Programme; RFL, Royal Free London NHS Foundation Trust’s North London Breast Screening Programme.

Using the calculation explained in the online supplemental file 1, the expected CDR increase for the XR workflow in a prospective setting would be 1.2 per 1000 cases, offsetting the CDR decrease of 0.1 per 1000 cases, found in the sIR and DRT simulations, to an expected CDR increase of 1.1 per 1000 cases in a combination workflow of sIR+XR or DRT+XR.

## Discussion

The ARIES study included more than 3 06 000 cases from different UK regions and diverse breast screening populations, enabling subgroup analyses across participant age, breast density and ethnicity. Non-inferiority tests passed for all outcome metrics on all assessed subgroups, and superiority tests passed for the majority of them. Differences within participant subgroups, with regard to the expected impact (ie, point estimates of the absolute differences between workflows with AI compared with without), were small, demonstrating important evidence towards the AI system’s safety and effectiveness. In addition, significant possible workload savings were modelled across all centres.

While most point estimates showed an improvement on every metric across all subgroups, the CDR estimates showed a decrease between −0.24 and −0.08 per 1000, across participant subgroups compared with standard DR. This is in line with the expectations from the specific workflows that are designed for operational improvements rather than improving cancer detection, as well as the retrospective study design that prevents the AI from detecting cancers in patients who have not been recalled for further assessment by human readers in the past.

In a prospective setting, additional cancer detection can be achieved by implementing the AI system as an XR, as published by Ng *et al*, where the AI flags cases for additional human review that have not been recalled in the DR process,^4^ and thus ideal for complementing the sIR and DRT workflows.

The IC flag rate, used as a proxy measure for additional cancer detection, was 41.2%, indicating the opportunity to increase CDR. Although flagging a case as suspicious does not guarantee that a cancer will indeed be picked up by the human readers, increasing CDR is a very realistic and likely scenario, as has been shown in recent publications.^3 4^ Moreover, in a previous retrospective study of the AI system evaluated in ARIES, the AI’s IC flag rate was 24.7% for 2-year ICs,^2^ and this translated into a CDR increase in live use ranging from 0.7 to 1.6 per 1000 cases after implementation at the same sites as an additional reader.^4^ In another retrospective study, the IC flag rate of 34.1% for 3-year ICs^19^ converted into a CDR increase of 1.0 per 1000 cases in a subsequent prospective evaluation.^20^ The method used to estimate the translation of IC flag rate in a retrospective study to CDR increase in a prospective setting for AI as an additional reader (online supplemental methods section) yields an expected CDR increase of 1.2 per 1000 cases.

This is the first study in breast screening to present a granular assessment of an AI system’s clinical impact, in terms of multiple subgroups and the use of various relevant clinical and operational outcome metrics. Several large-scale studies have been published, but without performing subgroup analyses.^2 21 22^ A large-scale study evaluating the same AI system assessed generalisability across different mammography equipment vendors (Hologic, GE, Siemens, IMS), but did not include the further subgroup analyses presented here.^2^ Others limited their report to one patient characteristic, such as age^23^ or breast density^24 25^ and SEN as a single outcome metric.^23^,^25^

The main limitations of the ARIES study are inherently linked with its retrospective nature, which is unavoidable given the large and heterogeneous population required to perform such extensive subgroup analyses. First, human reader breast density assessments at the individual patient level were not available for screening cases at the study centres, as it is not a part of standard screening practice in the UK. Using an automated breast density assessment tool, however, made it feasible to obtain a BIRADS density score for this large cohort of more than 306 000 screens. The breast density tool used was developed and validated on a large dataset from the USA,^16^ and the breast density distribution of the study screening population obtained from this tool was in line with distributions found elsewhere in Europe.^25^,^28^

Also, as follow-up duration was limited, the collection of the ICs is expected to be incomplete for the latter years of the study dataset. However, the follow-up duration used is nonetheless relevant for comparing subgroups and ensuring that within every subgroup, relevant cancers have been detected. In addition, ethnicity information was only available for analysis at one of the centres. This resulted in wider confidence intervals for the IC flag rate for the ethnicity subgroups, although the point estimates were in line with the other subgroups. Finally, use of AI as a second reader is expected to minimally impact human behaviour compared with concurrent use of AI for decision support, which would require confirmation prospectively.

The use of relevant metrics and subgroup analyses is key for meaningful evaluations that can inform the safety and effectiveness of AI and address an important area of AI bias.^5 29 30^ The non-inferiority, superiority results and expected impact on clinical and operational metrics (ie, point estimate differences between workflows with and without AI) across every participant subgroup in a large-scale and heterogeneous population provide confidence in the safe deployment and prospective use of the AI system. The study has shown how substantial workload savings with AI, supporting the sustainability of breast screening, can go hand-in-hand with safe performance across patient subgroups, ensuring screening quality for participants of all ages, breast densities and ethnicities.

## Supplementary material

10.1136/bmjhci-2024-101318 online supplemental file 1

## Data Availability

Data are available upon reasonable request.
