# Supplementary material for: Assessing artificial intelligence in breast screening with stratified results on 306 839 mammograms across geographic regions, age, breast density and ethnicity: A Retrospective Investigation Evaluating Screening (ARIES) study
Source: BMJ Health Care Inform. 2025 May 14;32(1):e101318. doi: 10.1136/bmjhci-2024-101318 (PMC12083354; doi:10.1136/bmjhci-2024-101318)
Supplement: online supplemental file 1 [file bmjhci-32-1-s001.pdf]

SUPPLEMENT

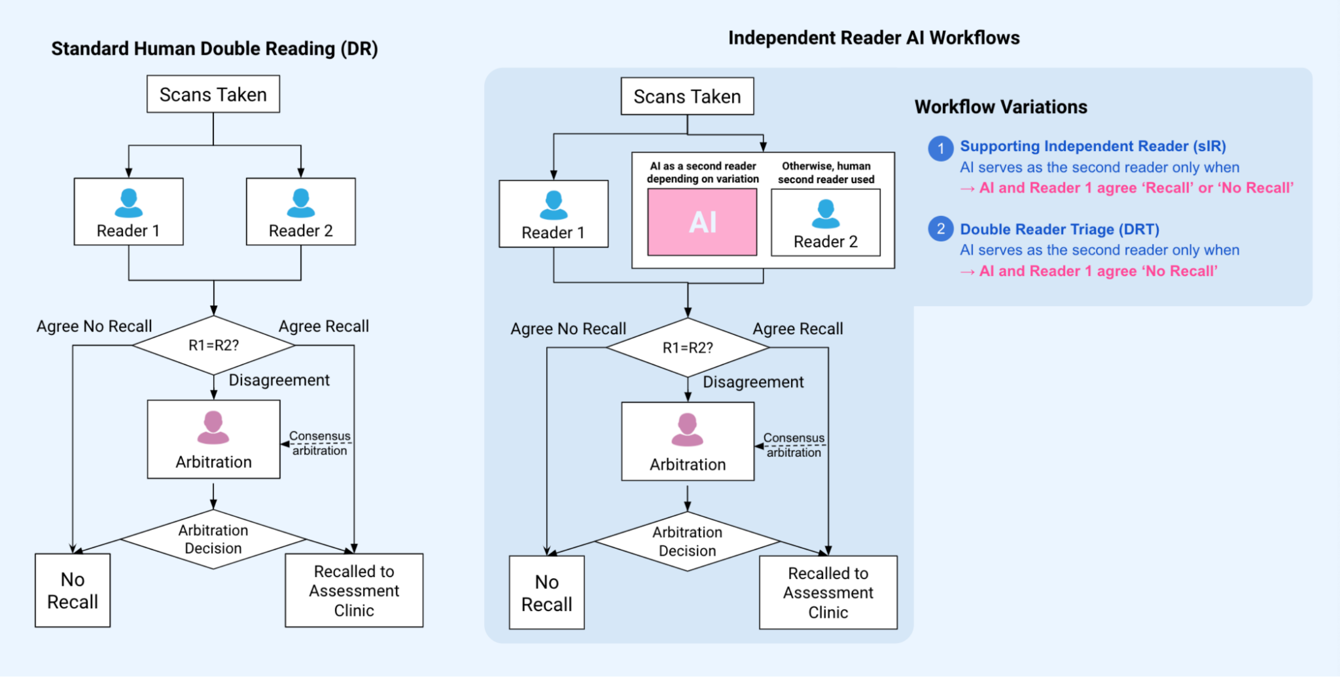

Supplemental Figure 1. Workflow diagrams.

In the left panel the standard human double reading process is shown. On the right the AI workflows used in the study are depicted.

Abbreviations: R = human reader

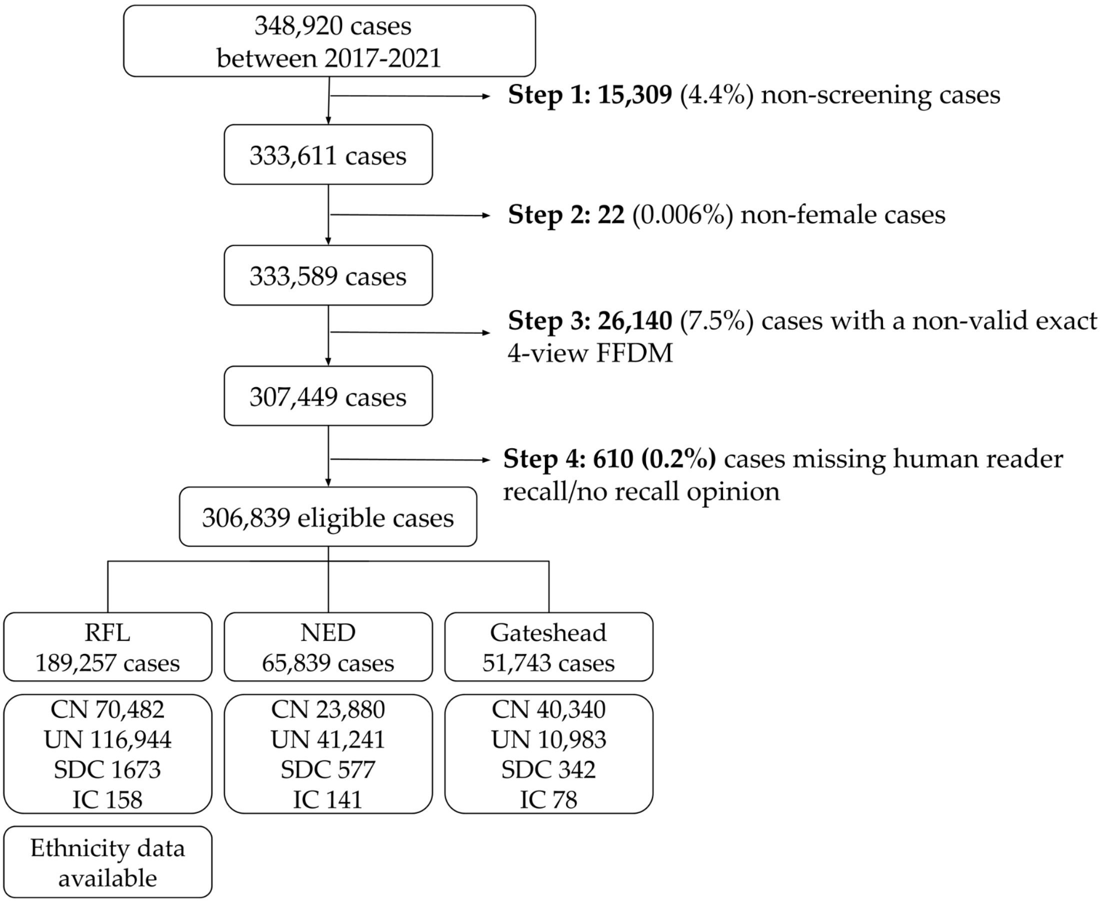

Supplemental Figure 2. STARD (Standards for Reporting of Diagnostic Accuracy Studies) diagram showing the exclusions applied in the study.

Abbreviations: CN = confirmed negative; UN = unconfirmed i.e. neither confirmed negative or cancer; SDC = screen-detected cancer; IC = interval cancer

SUPPLEMENTAL METHODS

Study Centres and Data

All centres included in the study participate in the UK NHSBSP and adhere to a three-year screening interval, inviting women between 50 and 70 years old. A small cohort of women between 47 and 49 years, who were eligible for the UK ageX trial (https://www.ceu.ox.ac.uk/research/agex-trial/agex-trial) were also included at NED and RFL. All centres acquired full-field digital mammograms (FFDM) for screening using Hologic (Selenia Dimensions or Lorad Selenia) mammography hardware equipment.

Patient and Public Involvement

Organised regular meetings were held with Kheiron Medical Technologies' Patient and Public Involvement (PPI) Board to identify the most relevant research topics and meaningful outcomes for the study, which revealed a clear interest in the performance of the AI system with regard to different subgroups. During the meetings, the study design and analysis in layperson's language were presented and discussed and the PPI board has continued to be updated about the progress of the project.

Confirmation and Setting of Operating Points

The output of the AI ensemble model is a number between 0 and 1, indicating a ranking of screens, according to how suspicious a screen is interpreted by the AI. Although the ordering is not changed, this raw output can be influenced by image characteristics, which are related to differences in mammography hardware machine type and software [1]. The operating points (OPs) of the AI system, however, are binary recommendations, set at a specified threshold that corresponds to a sensitivity-specificity trade-off. This can be either a balanced trade-off or trade-offs that emphasise sensitivity or specificity, enabling it to be used in various AI workflows that support different and varying clinical and operational outcomes. As these OPs

are usually based on threshold values, set on datasets from different mammography hardware machine vendors, models and software versions, confirmation or, in some cases, adaptation of the OPs is needed, before these can be used in a study or in clinical practice.

During the preparation phase of the ARIES study, a standard deployment procedure was executed to confirm appropriate decision thresholds for each AI operating point. The threshold assessment procedure was performed with a dataset disjoint from the trial dataset. The procedure was predetermined and prescribed in the study Data Management Plan. The thresholding and trial datasets were randomly split from each centre at a screening participant level to ensure both datasets were representative of the source screening population and to avoid that different screens of the same participant could be included in both datasets. It's important to note that the underlying machine learning ensemble model remained unchanged by this process.

Statistical analysis

Calculation of outcome measures

Cancers included screen-detected cancers and interval cancers, confirmed via biopsy or histo-pathology within 180 days or 1,095 days, respectively. Confirmed negative cases were confirmed with a 'three-year' negative follow-up result after the original screening date, with no evidence of malignancy in between. For cancer cases, if a case-wise recall was made by historical double reading or by double reading with AI, it was considered a true positive without further verification of the location or laterality of the confirmed cancer indicated. Outcome metrics were defined as follows:

Cancer detection rate (CDR): number of cancers detected divided by the number of all cases

Positive predictive value (PPV): number of cancers recalled divided by the number of recalled cases

Recall rate (RR): number of cases recalled divided by the number of all

	cases
Arbitration rate (AR):	number of arbitrations conducted divided by the number of all cases
Sensitivity (SEN):	number of recalled cancers divided by the number of all known cancer cases
Specificity (SPEC):	number of confirmed negatives not recalled divided by the number of all confirmed negative cases.

Statistical testing

All non-inferiority and superiority tests used an alpha of 0.05 and a relative non-inferiority margin of 5%.

The ratio between a double reading metric and the AI workflow metric was calculated by dividing the AI workflow metric by the double reading metric. A bootstrap procedure which consisted of 200,000 iterations was executed and for each iteration, the ratios were calculated. Subsequently, these were used to create percentile-based confidence intervals. Each bootstrap contained the same number of cases as the initial dataset. The samples were drawn with replacement.

All analyses for breast density subgroups were done post-hoc under further research. The non-inferiority and superiority tests on age and ethnicity subgroups were performed post-hoc. Although all AI operating points were defined and fixed prior to the AI system's analysis of any study data, the modelling of DRT presented is post-hoc as it used a higher-specificity operating point than originally defined, in alignment with an update in the tool's intended use after commencement of the study. Tests for AR were also added as post-hoc for completeness. All other tests presented were defined in the study protocol and SAP. If information on a patient characteristic was missing, the case was only excluded for the analysis of the subgroup considering that specific patient characteristic.

All data was analysed using Python version 3.11.

Calculation of additional cancer detection opportunity with the AI additional reader workflow

In the AI additional reader (or XR) workflow, the screens of patients who have not been recalled for further assessment in the human double reading process, are read by the AI. If the AI indicates a case as suspicious an extra human review takes place and this offers the opportunity to recall a case. This AI workflow is specifically designed to increase the cancer detection rate.

In a previous retrospective study of the AI system evaluated in ARIES, encompassing the same machine learning model [2], the interval cancer case flag rate for the MaMMaKlinika centre in Hungary was 24.7%. Implementing the same AI system as an additional reader in a prospective live setting at the same centre, yielded an increase in CDR ranging from 0.7 to 1.6 per 1,000 cases [3].

A retrospective study in Scotland, in the iCAIRD project, showed an interval cancer case flag rate of 34.1% for the AI system evaluated in ARIES [1]. A subsequent prospective service evaluation, called GEMINI, of the same AI system as an additional reader resulted in a CDR increase of 1.0 per 1,000 cases (unpublished, presented at European Conference of Radiology (ECR) 2024, Vienna).

From these prospective evaluation results, every 10% of interval cancer flag rate corresponds to a 0.28-0.3/1000 CDR increase achieved. Using these results in a linear regression analysis, makes it possible to obtain a regression coefficient (0.003), which can then be used to estimate the expected CDR increase proportion, based on an interval cancer case flag proportion.

$$\Delta CDR_{expected} = 0.003 * \text{IC flag rate}$$

Applying this formula in the ARIES study yields an expected CDR increase of 1.2 per 1,000 cases.

SUPPLEMENTAL RESULTS FOR OUTCOME METRICS

The results for AR for sIR and DRT compared to DR show a decrease ranging from -1.4% to -0.49% across subgroups (Supplementary Table S1). The results for SEN are in alignment with the results for CDR, while the results for SPEC are in alignment with the results for RR in terms of the impact of sIR and DRT to DR across subgroups (Supplementary Table S1, manuscript Tables 3 and 4).

The confidence intervals for the absolute differences between double reading with and without AI are presented in Supplementary Tables S2 and S3 for clinical (CDR, PPV) and operational metrics (RR), respectively. The confidence intervals are percentile-based, formulated using a bootstrapping procedure with 100,000 iterations.

DATA AVAILABILITY STATEMENT

Subject to patient privacy and confidentiality obligations, access to patient-level breast screening data, used in the study, and supporting clinical information can be made available upon request and subject to information governance at the participating sites. Data access requests will be processed within four weeks. Such requests can be made to the corresponding author by email.

CODE AVAILABILITY STATEMENT

The code used for training and deploying the evaluated AI system has a large number of dependencies on internal tooling, proprietary components, infrastructure and hardware. The full code release is therefore not feasible. We provide a technical description of the AI system in the online Methods section together with a code repository to facilitate reproducibility of research involving deep learning models for breast cancer detection in digital mammography. The code provided under https://github.com/Kheiron-Medical/mammo-net demonstrates the training and testing of state-of-the-art convolutional neural networks which build the core component of most commercially available breast cancer AI systems.

REFERENCES

1 de Vries CF, Colosimo SJ, Staff RT, *et al.* Impact of Different Mammography Systems on Artificial Intelligence Performance in Breast Cancer Screening. *Radiol Artif Intell*. 2023;5:e220146.

2 Sharma N, Ng AY, James JJ, *et al.* Multi-vendor evaluation of artificial intelligence as an independent reader for double reading in breast cancer screening on 275,900 mammograms. *BMC Cancer*. 2023;23:460.

3 Ng AY, Oberije CJG, Ambrózay É, *et al.* Prospective implementation of AI-assisted screen reading to improve early detection of breast cancer. *Nat Med*. 2023;29:3044–9.

Supplemental Table S1. Additional results per subgroup – Sensitivity and specificity

Subgroup		Standard Human Double Reading (DR)	Supporting Independent Reader (sIR)			Double Reader Triage (DRT)		
			Value (95% CI)	Absolute Difference	Test Passed	Value (95% CI)	Absolute Difference	Test Passed
**Sensitivity (SEN) (%)**								
Centre	RFL	92.0 (90.6-93.1)	90.0 (88.5-91.3)	-2.0	Non-inferiority	90.3 (88.9-91.6)	-1.6	Non-inferiority
	NED	81.6 (78.6-84.3)	80.9 (77.9-83.6)	-0.70	Non-inferiority	80.9 (77.9-83.6)	-0.70	Non-inferiority
	Gateshead	81.7 (77.7-85.1)	80.5 (76.4-84.0)	-1.2	Non-inferiority	80.5 (76.4-84.0)	-1.2	Non-inferiority
Breast Density	A/B	90.2 (88.6-91.6)	88.6 (86.9-90.1)	-1.6	Non-inferiority	89.1 (87.4-90.6)	-1.1	Non-inferiority
	C/D	79.4 (77.2-81.4)	78.4 (76.2-80.4)	-1.0	Non-inferiority	78.2 (76.0-80.2)	-1.2	Non-inferiority
Age	<60 years	79.4 (77.1-81.5)	78.4 (76.0-80.6)	-1.1	Non-inferiority	78.0 (75.6-80.2)	-1.4	Non-inferiority
	≥60 years	88.9 (87.3-90.3)	87.5 (85.8-89.0)	-1.4	Non-inferiority	87.9 (86.2-89.4)	-1.0	Non-inferiority
Ethnicity	White	91.3 (89.4-92.9)	89.1 (87.1-90.9)	-2.2	Non-inferiority	89.4 (87.4-91.2)	-1.9	Non-inferiority
	Non-White	92.6 (90.1-94.4)	91.1 (88.5-93.2)	-1.4	Non-inferiority	91.3 (88.7-93.4)	-1.2	Non-inferiority
**Specificity (SPEC) (%)**								
Centre	RFL	95.6 (95.4-95.7)	96.1 (95.9-96.2)	+0.50	Superiority	96.0 (95.9-96.2)	+0.46	Superiority
	NED	95.9 (95.6-96.1)	96.3 (96.0-96.5)	+0.35	Superiority	96.4 (96.2-96.7)	+0.53	Superiority
	Gateshead	97.4 (97.1-97.7)	97.6 (97.3-97.8)	+0.17	Superiority	97.6 (97.3-97.9)	+0.22	Superiority
Breast Density	A/B	96.8 (96.6-96.9)	97.1 (97.0-97.2)	+0.34	Superiority	97.1 (97.0-97.2)	+0.35	Superiority
	C/D	95.7 (95.5-95.8)	96.0 (95.8-96.2)	+0.34	Superiority	96.1 (95.9-96.3)	+0.47	Superiority
Age	<60 years	95.9 (95.7-96.0)	96.2 (96.1-96.4)	+0.38	Superiority	96.3 (96.2-96.5)	+0.46	Superiority
	≥60 years	96.9 (96.7-97.1)	97.2 (97.0-97.4)	+0.28	Superiority	97.2 (97.1-97.4)	+0.32	Superiority
Ethnicity	White	95.5 (95.3-95.7)	96.0 (95.8-96.2)	+0.51	Superiority	96.0 (95.8-96.2)	+0.47	Superiority
	Non-White	95.7 (95.5-96.0)	96.2 (96.0-96.5)	+0.50	Superiority	96.2 (95.9-96.4)	+0.40	Superiority
**Arbitration Rate (AR) (%)**								
Centre	RFL	7.5 (7.3-7.6)	6.5 (6.4-6.6)	-0.96	Superiority	6.6 (6.5-6.7)	-0.89	Superiority

	NED	3.1 (3.0-3.3)	1.7 (1.6-1.8)	-1.4	Superiority	2.1 (2.0-2.2)	-0.99	Superiority
	Gateshead	1.8 (1.7-2.0)	1.1 (1.0-1.2)	-0.76	Superiority	1.3 (1.2-1.4)	-0.49	Superiority
Breast Density	A/B	3.6 (3.5-3.7)	2.7 (2.6-2.8)	-0.91	Superiority	2.9 (2.8-3.0)	-0.70	Superiority
	C/D	4.9 (4.7-5.0)	3.6 (3.5-3.7)	-1.3	Superiority	3.9 (3.8-4.0)	-0.93	Superiority
Age	<60 years	4.7 (4.6-4.8)	3.5 (3.4-3.6)	-1.2	Superiority	3.8 (3.7-3.9)	-0.90	Superiority
	≥60 years	3.5 (3.4-3.6)	2.6 (2.5-2.7)	-0.91	Superiority	2.8 (2.7-2.9)	-0.67	Superiority
Ethnicity	White	7.4 (7.2-7.5)	6.4 (6.3-6.6)	-0.95	Superiority	6.5 (6.3-6.6)	-0.89	Superiority
	Non-White	6.7 (6.5-6.9)	5.8 (5.7-6.0)	-0.85	Superiority	5.9 (5.7-6.1)	-0.78	Superiority

Supplemental Table S2. Additional results for clinical metrics per subgroup – confidence intervals for the absolute difference between double reading with and without AI

Subgroup		Standard Human Double Reading (DR)	Supporting Independent Reader (sIR)		Double Reader Triage (DRT)	
			Value (95% CI)	Absolute Difference (95% CI)*	Value (95% CI)	Absolute Difference (95% CI)*
**Cancer Detection Rate (CDR) per 1000**						
Centre	RFL	8.9 (8.5-9.3)	8.7 (8.3-9.1)	-0.19 (-0.25, -0.13)	8.7 (8.3-9.2)	-0.16 (-0.22, -0.11)
	NED	8.9 (8.2-9.6)	8.8 (8.1-9.6)	-0.08 (-0.18, +0.03)	8.8 (8.1-9.6)	-0.08 (-0.15, -0.02)
	Gateshead	6.6 (6.0-7.4)	6.5 (5.9-7.3)	-0.10 (-0.21, +0.02)	6.5 (5.9-7.3)	-0.10 (-0.19, -0.02)
Age	<60 years	6.2 (5.8-6.6)	6.1 (5.7-6.5)	-0.08 (-0.16, -0.01)	6.0 (5.7-6.4)	-0.11 (-0.17, -0.06)
	≥60 years	10.2 (9.7-10.7)	10 (9.5-10.6)	-0.16 (-0.25, -0.08)	10.1 (9.6-10.6)	-0.11 (-0.17, -0.06)
Breast Density	A/B	7.6 (7.2-8.0)	7.5 (7.1-7.9)	-0.13 (-0.20, -0.08)	7.5 (7.1-7.9)	-0.09 (-0.14, -0.05)
	C/D	8.9 (8.4-9.4)	8.8 (8.3-9.3)	-0.10 (-0.21, +0.01)	8.7 (8.2-9.3)	-0.14 (-0.21, -0.07)
Ethnicity	White	9.9 (9.3-10.5)	9.6 (9.0-10.3)	-0.24 (-0.35, -0.14)	9.7 (9.1-10.3)	-0.21 (-0.30, -0.12)
	Non-White**	8.0 (7.4-8.7)	7.9 (7.3-8.6)	-0.12 (-0.22, -0.05)	7.9 (7.3-8.6)	-0.11 (-0.20, -0.03)
**Positive Predictive Value (PPV) (%)**						
Centre	RFL	17.7 (16.9-18.5)	19.1 (18.3-20.0)	+1.4 (+1.27, +1.61)	19.1 (18.2-19.9)	+1.4 (+1.19, +1.51)
	NED	17.9 (16.7-19.3)	19.1 (17.7-20.5)	+1.1 (+0.82, +1.46)	19.7 (18.3-21.2)	+1.7 (+1.48, +2.01)
	Gateshead	20.2 (18.3-22.2)	20.1 (18.3-22.1)	-0.05 (-0.48, +0.38)	21.1 (19.2-23.2)	+1.0 (+0.63, +1.29)
Age	<60 years	12.8 (12.1-13.5)	13.4 (12.7-14.2)	+0.60 (+0.40, +0.81)	13.7 (13.0-14.5)	+0.90 (+0.73, +1.07)
	≥60 years	26.2 (25.1-27.4)	27.4 (26.2-28.6)	+1.2 (+0.83, +1.53)	28.1 (26.9-29.3)	+1.9 (+1.62, +2.16)
Breast Density	A/B	19.5 (18.6-20.4)	20.5 (19.6-21.5)	+1.0 (+0.77, +1.26)	21.0 (20.0-22.0)	+1.5 (+1.23, +1.67)

	C/D	17.6 (16.7-18.5)	18.2 (17.3-19.2)	+0.65 (+0.35, +0.94)	18.8 (17.9-19.8)	+1.2 (+1.03, +1.45)
Ethnicity	White	19.6 (18.5-20.7)	21.1 (19.9-22.4)	+1.6 (+1.29, +1.82)	21.1 (19.9-22.3)	+1.5 (+1.22, +1.72)
	Non-White**	17.5 (16.2-18.9)	19.0 (17.6-20.5)	+1.5 (+1.20, +1.77)	18.9 (17.4-20.4)	+1.4 (+1.10, +1.63)

Abbreviations: CI = confidence interval;

* Percentile based 95% CI with bootstrapping

** Non-white is a combination of Black, Asian and Mixed/other ethnicity

Supplemental Table S3. Additional results for operational metric Recall Rate overall and per centre – confidence intervals for the absolute difference between double reading with and without AI

Subgroup	DR	Supporting Independent Reader (sIR)		Double Reader Triage (DRT)	
	Value (95% CI)	Value (95% CI)	Absolute Difference (95% CI)*	Value (95% CI)	Absolute Difference (95% CI)*
**Recall Rate (%)**					
Overall	4.4 (4.4-4.5)	4.1 (4.1-4.2)	-0.28 (-0.32, -0.25)	4.1 (4.0-4.1)	-0.37 (-0.39, -0.35)
RFL	5.0 (4.9-5.1)	4.5 (4.5-4.6)	-0.48 (-0.51, -0.45)	4.6 (4.5-4.7)	-0.44 (-0.47, -0.41)
NED	5.0 (4.8-5.1)	4.6 (4.5-4.8)	-0.34 (-0.40, -0.27)	4.5 (4.3-4.6)	-0.48 (-0.53, -0.42)
Gateshead	3.3 (3.1-3.4)	3.2 (3.1-3.4)	-0.04 (-0.09, +0.01)	3.1 (2.9-3.2)	-0.20 (-0.23, -0.16)

Abbreviations: DR = standard human double reading; CI = confidence interval

* Percentile based 95% CI with bootstrapping
